# Detection of intramural scar by electroanatomic mapping versus MRI in patients with non-ischemic cardiomyopathy

**DOI:** 10.1186/1532-429X-15-S1-P65

**Published:** 2013-01-30

**Authors:** Benoit Desjardins, Fred Morady, Frank Bogun

**Affiliations:** 1 Department of Radiology, University of Pennsylvania Medical Center, Philadelphia, PA, USA; 2 Medicine, University of Michigan, Ann Arbor, MI, USA

## Background

Ventricular arrhythmias have been described to originate from intramural locations. Intramural scar can be assessed by delayed enhanced magnetic resonance imaging (DE-MRI ). The objective of this study was to determine whether endocardial voltage mapping by catheter can detect the presence and extent of scar deep within the myocardial wall thickness, using DE-MRI as gold standard.

## Methods

**Figure 1:**
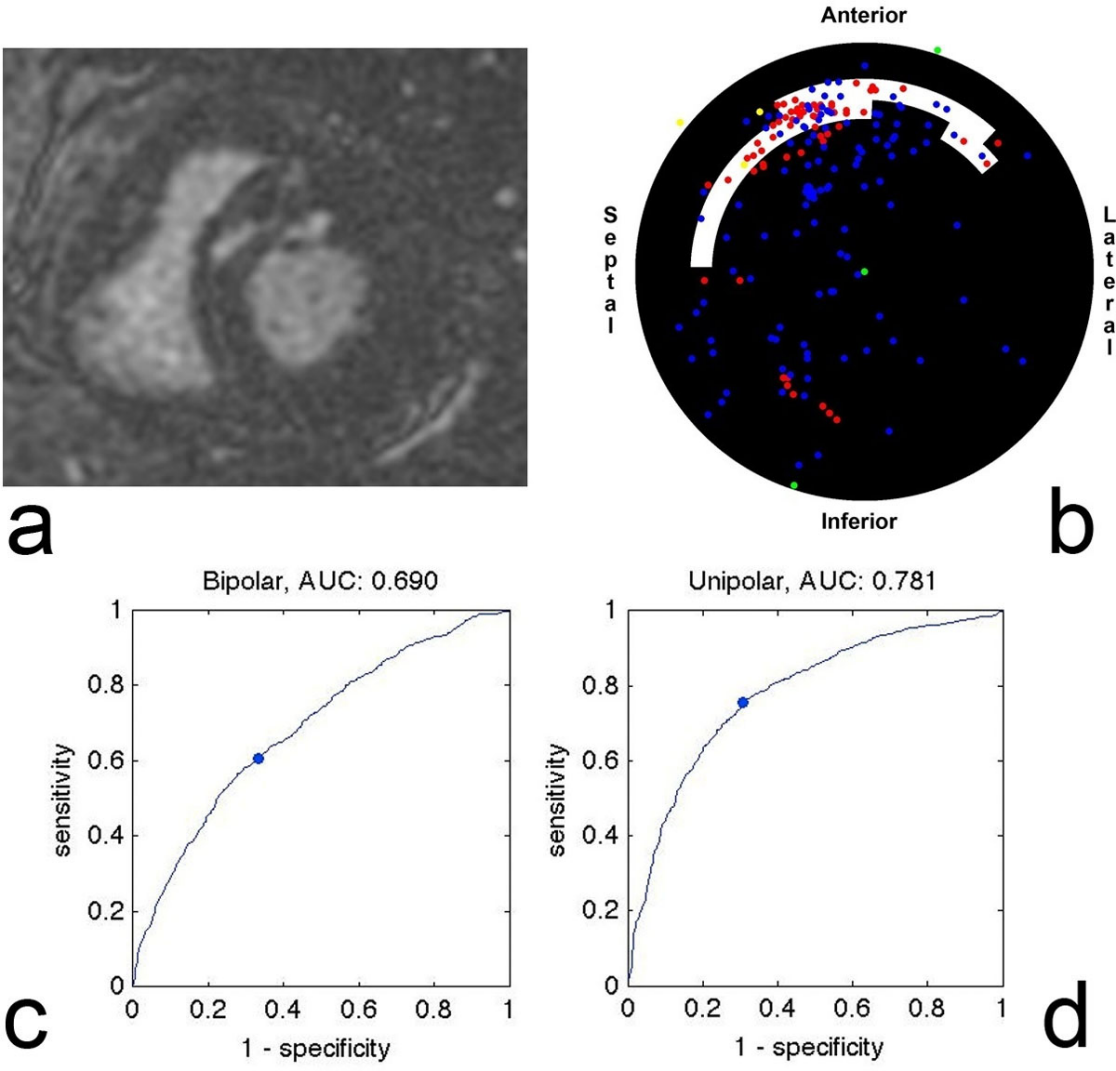


In 15 consecutive patients with structural heart disease and without contraindication to MRI, intramural scar was detected by DE-MRI (Fig 1a). All patients underwent endocardial voltage mapping by catheter. Both unipolar and bipolar voltage maps were constructed from the endocardium. The scar on DE-MRI was registered with the electroanatomic map using fiducials (apex, aorta, mitral valve) and a polar representation of the corresponding endocardial voltage was generated (Fig 1b: red points: low voltage, purple points: high voltage, yellow and green points: fiducials).

## Results

On MRI, scar volume was 11.7±8.0 cm3, endocardial scar area was 19.3±13.4 cm2. Scar thickness was 4.6±0.7 mm (39±7% of wall thickness), with a subendocardial rim thickness of 3.3±1.6 mm (26±13%) and a subepicardial rim thickness of 4.8±2.6 mm (37±15%). Endocardial bipolar voltage measurements were 1.62±1.73 mV over the scar, 2.12±2.15 mV in a 1cm neighborhood around the scar, and 2.83±2.34 mV in the more remote myocardium (p<0.0001). Endocardial unipolar voltage measurements were 4.94±3.25 mV over the scar, 6.59±3.81 mV in a 1cm neighborhood around the scar, and 8.32±3.39 mV in the more remote myocardium (p<0.0001). Using ROC curves (Fig 1c,d) of the voltage maps, a unipolar cut-off value of 6.78 mV (AUC 0.78) and a bipolar cut-off value of 1.55 mV (AUC 0.69) best separated endocardial measurements overlying scar from the endocardial measurements more remote to the scar.

## Conclusions

Although scar deep within the myocardial wall is far from the tip of the endocardial measurement catheter, its presence and extent can still be detected by endocardial electroanatomic mapping using a combination of unipolar and bipolar voltage, with unipolar voltage being more useful.

## Funding

NIH K23 EB006481

